# Real-Time Life-Cycle Monitoring of Composite Structures Using Piezoelectric-Fiber Hybrid Sensor Network

**DOI:** 10.3390/s21248213

**Published:** 2021-12-08

**Authors:** Yinghong Yu, Xiao Liu, Jiajia Yan, Yishou Wang, Xinlin Qing

**Affiliations:** School of Aerospace Engineering, Xiamen University, Xiamen 361105, China; yuyinghong@stu.xmu.edu.cn (Y.Y.); liuxiao@stu.xmu.edu.cn (X.L.); yanjiajia@xmu.edu.cn (J.Y.); wangys@xmu.edu.cn (Y.W.)

**Keywords:** composite structures, structural health monitoring, hybrid sensor network, progress of reaction, probability diagnostic imaging

## Abstract

In this paper, an in situ piezoelectric-fiber hybrid sensor network was developed to monitor the life-cycle of carbon fiber-reinforced plastics (CFRPs), from the manufacturing phase to the life in service. The piezoelectric lead-zirconate titanate (PZT) sensors were inserted inside the composite structures during the manufacturing process to monitor important curing parameters, including the storage modulus of resin and the progress of the reaction (POR). The strain that is related to the storage modulus and the state of resin was measured by embedded fiber Bragg grating (FBG) sensors, and the gelation moment identified by the FBG sensors was very close to those determined by dynamic mechanical analysis (DMA) and POR. After curing, experiments were conducted on the fabricated CFRP specimen to investigate the damage identification capability of the embedded piezoelectric sensor network. Furthermore, a modified probability diagnostic imaging (PDI) algorithm with a dynamically adaptive shape factor and fusion frequency was proposed to indicate the damage location in the tested sample and to greatly improve the position precision. The experimental results demonstrated that the average relative distance error (RDE) of the modified PDI method was 68.48% and 46.97% lower than those of the conventional PDI method and the PDI method, respectively, with an averaged shape factor and fusion frequency, indicating the effectiveness and applicability of the proposed damage imaging method. It is obvious that the whole life-cycle of CFRPs can be effectively monitored by the piezoelectric-fiber hybrid sensor network.

## 1. Introduction

Carbon fiber-reinforced plastics (CFRPs) have gained increasing applications in the aerospace and automobile fields due to their extraordinary performance such as high specific strength and stiffness, corrosion resistance, and strong designability [1,2]. Thermoset CFRPs are commonly manufactured by stacking prepregs, and then heat and pressure are applied to them to cure the resin and exclude air voids [3]. For mass production of composite materials, vacuum bag molding technology is gaining interest due to its simplicity, cost competitiveness, and short production time. However, the curing reaction of the resin is complicated, especially for co-cured large-scale composite materials, and can potentially induce molding defects, such as dry spots, voids, or delamination, along with the mechanical property reduction of the final parts induced by improper manufacturing conditions, such as cure temperature and preservation time [4,5,6]. Borrero et al. [7] compared five curing conditions on the durability and compressive strength of concrete to recommend the most effective curing conditions on concrete’s characteristics. Daneshvar et al. [8] investigated the dynamic behavior of five small-scale FRP-strengthened corroded steel-reinforced concrete (RC) slabs experimentally and numerically. The experimental results showed that FRP strengthening helps to improve the dynamic performance of the corroded plate. With the increasing demand for the performance of composite structures, many structural health monitoring (SHM) methods are required to be incorporated into life-cycle monitoring plans to obtain higher optimal quality [9]. Accordingly, a number of sensing and measurement technologies have emerged such as optical fiber sensing [10,11,12,13], ultrasonic sensing [14,15,16,17], electromechanical impedance [18,19,20], thermometers [21,22], and pressure transducers [23,24]. According to these sensing technologies, they enable one to follow manufacturing parameters such as the progress of the reaction, the residual stress, the development of the resin complex modulus, and the events during the curing cycle such as gelation and vitrification. To monitor the curing parameters, ultrasonic testing is an effective method, which is sufficiently sensitive to structural status changes. Koissin et al. [25] adopted a nonlinear ultrasonic immersion technique to monitor the resin curing in an aluminum-adhesive-aluminum laminate. Khadka et al. [26] embedded a fiber Bragg grating (FBG) sensor and thermocouple in an epoxy system cured at 12 °C to determine its curing characteristics. However, they neglected the impact of the fiber on the resin, which would lead to inaccurate measurement results. The actual trend in research deals with the application of the embedded sensor network to perform in situ monitoring, with real-time and auto-checking abilities [27]. The embedded smart system is beneficial to reveal the complex physical phenomena inside materials and can be integrated permanently with the molded composite materials to provide information about the structural performance changes during its whole life-cycle. For achieving life-cycle monitoring of composite structures, Eum et al. [28] used optical fiber sensors to monitor both the manufacturing process and the strain of composite structures for quality assurance and integrity assessment. Furthermore, Minakuchi et al. [27] performed life-cycle monitoring of a large-scale CFRP by fiber-optic-based distributed sensing. However, these measurement methods stated above pose the problem of localized sensing and the limitations of curing parameter monitoring. In contrast to these sensing technologies, piezoelectric sensors have a large-scale sensing ability due to their long-distance propagation and can achieve in situ process monitoring and in-service inspection based on ultrasonic propagation.

Therefore, a piezoelectric-fiber hybrid sensor network was proposed to monitor more curing parameters and improve the reliability and accuracy of the monitoring results during the curing process, and it can be further used to achieve online health monitoring when it is in service. In the hybrid sensor network, PZT sensors encapsulated in the Stanford Multiactuator-Receiver Transduction (SMART) are used to monitor the storage modulus of resin and the progress of reaction of a large-area structure, and it can be integrated with the composite structures after curing. The distributed FBG sensors were used to measure the strain during the curing process and to identify the moment of gelation in the curing reaction to verify the correctness of the ultrasonic measurements.

For service inspection of the molded composite structures, guided waves-based structural health monitoring is a promising method because of the large monitoring area, relatively low attenuation, and high execution efficiency [29,30,31]. In a typical guided waves-based SHM system, the PZT sensors are the most commonly used to stimulate and receive guided waves for damage identification. The sensor layer (that is SMART layer) with a PZT sensor network was embedded into the composite laminate during the fabrication. It can serve as an active way to provide detailed information about the structural status changes and to realize damage assessment with high accuracy. In addition to damage detection, many researchers are dedicated to imaging methods to locate damage more intuitively and efficiently. Among the damage imaging methods, the probability-based diagnostic imaging (PDI) algorithm is the most commonly used method due to its conceptual simplicity, easy implementation, and no requirement about the prior knowledge of guided waves [32,33,34]. In the PDI method, the shape factor is a parameter used to control the affected area, which is determined empirically and remains constant for all actuator-sensor paths. Improper shape factors may lead to inaccurate or even wrong imaging results when using the conventional PDI method [35,36]. Consequently, it is highly required to propose a variable shape factor to adjust the affected area of each sensing path and enhance the imaging precision of the PDI method. In this paper, a dynamically adaptive shape factor was proposed to adjust the affected area of all sensing paths in real-time based on the damage index. The experimental results showed that the damage location ability can be improved significantly by the modified PDI method.

Cure monitoring in the manufacturing stage and damage identification in the service stage are two vital parts in the whole life-cycle monitoring of CFRPs. In this paper, a piezoelectric-fiber hybrid sensor network was developed to determine the initial quality and assess the structural integrity of composite structures. The first wave packet of guided waves was extracted to monitor the storage modulus and loss factor of the resin and the progress of the reaction in order to better determine the curing conditions. The FBG sensors were used to measure the strain and identify the gelation moment. Another positive point is that the embedded PZT sensor network remains operational after demolding, so it can perform in situ and real-time SHM for the fabricated specimen. Furthermore, a modified PDI method with a dynamically adaptive shape factor was developed to improve the damage location accuracy effectively. The experimental results indicated that the piezoelectric-fiber hybrid sensor network can realize the life-cycle monitoring of composite materials and provide important technical support to realize the intelligence of composite materials.

## 2. Cure Monitoring

### 2.1. Experimental Setup for Cure Monitoring

**Figure 1:**
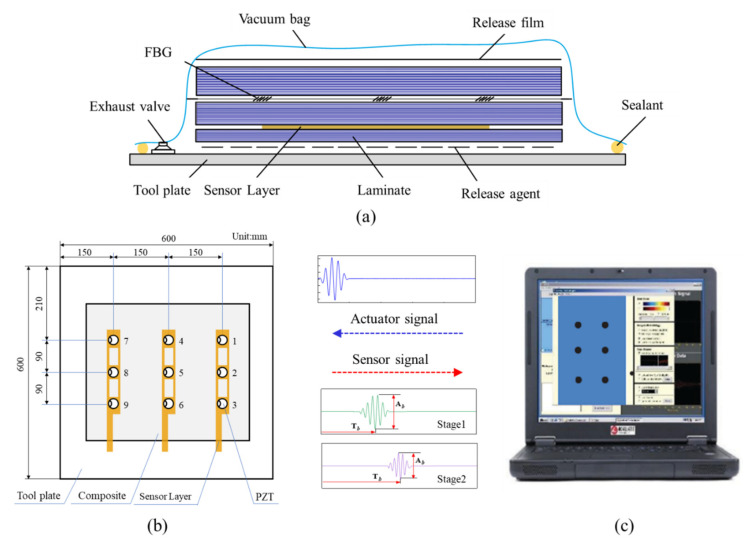
The experimental setup for cure monitoring based on ultrasonic guided waves. (**a**) Schematic diagram of prepreg molding process based on the VBM technology. (**b**) The detailed sensor layout. (**c**) Guided waves measurement system.

**Figure 2:**
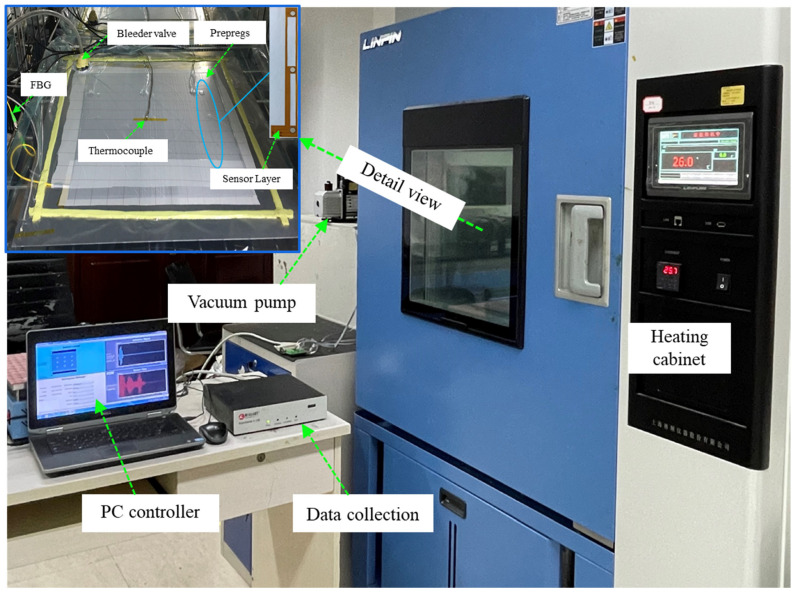
The schematic of the experiment platform for cure monitoring.

Figure 1 shows the experimental setup for guided waves measurement on the preform during the curing process. The experimentally fabricated specimen used in the experiment was an eight-layer unidirectional CFRP prepreg with a size of 400 mm × 400 mm × 1.835 mm, which is shown in Figure 1b. A breather was placed on the top of the CFRP to ensure even airflow, and then the release film and the vacuum bag were placed on the breather sequentially. It was known in advance that the CFRP was wrapped easily when cured with the vacuum bag molding (VBM) technology without a tool plate, which would affect the measurement accuracy and the molded part performance. Therefore, a 6061-aluminum plate with a size of 600 mm × 600 mm × 2 mm was adopted and the CFRP plate was placed on the tool plate. In the experiment, three sensor layers with nine PZT sensors were inserted between the first layer and the second layer of the specimen. Studies have shown that under certain conditions, the embedded sensor layer would not degrade the integrity of the host structure, by introducing a piezoelectric elements network supported on a thin flexible printed circuit substrate. The size of the PZT transducers was ϕ8 mm × 0.33 mm and their relative properties are described in Table 1. The distance between the actuator and the sensor was 150 mm. Moreover, one atmospheric pressure was applied to the stacked prepregs so that the transducers had good contact with the tested material, allowing better propagation of the guided wave through the aluminum plate. The scene picture of the experiment is shown in Figure 2. It can be found that the embedded sensor layer was hollowed out to minimize the damage to the CFRP plate. During the curing process, the transmitter was excited by a five-cycle Hamming windowed sinusoidal waveform supplied by Scan Genie II. Measurements of the receivers were also acquired by the system. The excitation voltage was 75 V and the signals were acquired by performing a frequency scan over each of the sensing paths. Upon the above experimental setup, the CFRP plate was gradually cured and guided wave signals of all sensing paths were recorded at regular intervals.

### 2.2. DMA Test

**Figure 3:**
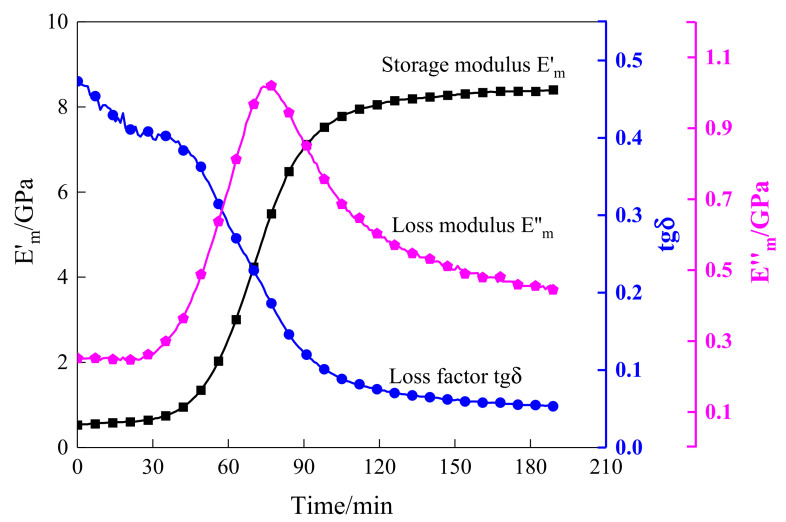
DMA testing curves of T300 prepregs.

Conventionally, the dynamic mechanical analysis (DMA) test is a common method used to accurately measure the dynamic storage modulus and loss modulus of resin in the curing process. In this paper, the specimens were tested with the DMA test under double cantilever support, and the temperature rose and remained at 10 °C with a periodic oscillating force. The curing behavior of composites characterized by the DMA test is shown in Figure 3. As can be seen from Figure 3, the DMA results contained the variation curve of the storage modulus $E_{m}{}^{\prime }$, loss modulus $E_{m}{}^{\prime \prime }$, and loss factor tgδ. The storage modulus is relative to the degree of cure (DOC), and it increased as the resin cured and arrived at the final value of approximately 8.4 GPa. Therefore, this parameter has the capability to identify several crucial steps including the gel point and vitrification point. On the contrary, the loss factor, the specific value of $E_{m}{}^{\prime }/E_{m}{}^{\prime \prime }$, showed a decreasing trend as the cure continued, and it eventually tended to a minimum value of about 0.053. This parameter can be used to estimate the energy variation throughout the curing process when the experimental specimen was cured at a constant temperature of 105 °C. The intersection of the loss factor and storage modulus curves is the point with the largest slope. At this point, the decreasing rate of the loss factor and the increasing rate of the storage modulus of the resin was fastest, indicating that the curing rate of the resin reached the maximum. The parameter of $E_{m}{}^{\prime \prime }$ indicates the energy loss of irreversible deformation in the curing process. It can be found that it was not positively correlated with time. It increased gradually with time for the first approximately 75 min until it reached a local maximum. Then, the loss modulus decreased with time until it reached a minimum value.

### 2.3. Results and Discussion of the Cure Monitoring Process

As mentioned above, the DMA test can represent the dynamic characteristics of resin in the curing process but poses the problem of not real-time measurements and the neglect of the impact of environmental factors on the results. The actual trend in research deals with the application of in situ smart sensors, with real-time acquisition capability. Due to the flexibility and reliability of the guided wave-based method, it can facilitate in situ and real-time cure monitoring of carbon fiber-reinforced composite plates manufactured by the VBM technology.

**Figure 4:**
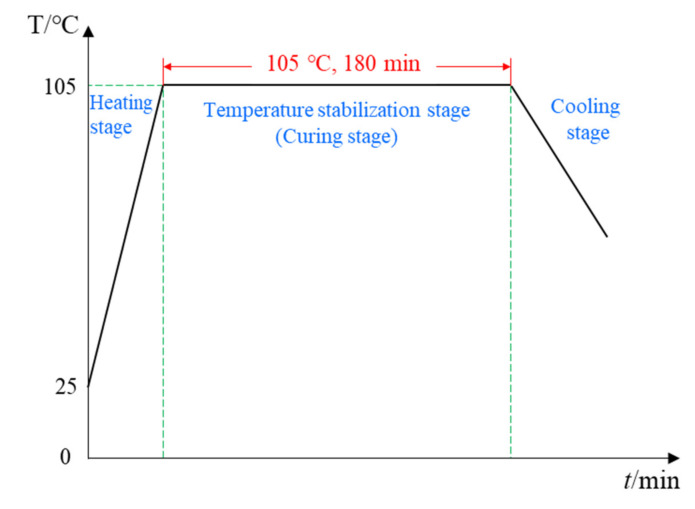
Recommended heating profile of the T300 prepreg provided by the manufacturer.

**Figure 5:**
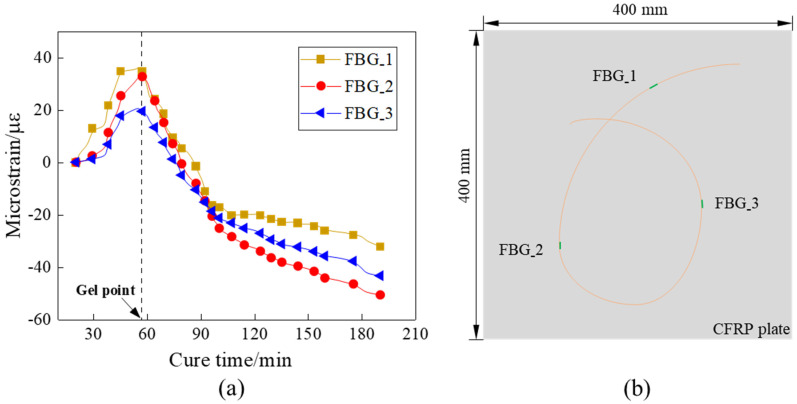
(**a**) The strain field of FBG sensors. (**b**) Schematic diagram of FBG sensors layout.

After the experimental preparation was completed, the regrading preform was placed into a heating cabinet for curing, and the recommended curing temperature profile is shown in Figure 4. The entire experiment process was carried out at a constant temperature of 105 ° C for 3 h. In the heating-up stage, the temperature rose from room temperature to 105 °C in the first 20 min, and then the temperature remained at 105 °C. Preceding studies have shown that the temperature and degree of cure field were evenly distributed in the curing process for thin CFRP plates (less than 2 mm), and the gradient of temperature and degree of cure can be ignored in the thickness scale. Therefore, the strain reduction is attributable to the chemical shrinkage of the resin rather than the exothermic reaction in the curing process. The strain field shown in Figure 5a was monitored by the distributed FBG sensor with three sensing points shown in Figure 5b, and the measurement of the strain signals was from the isothermal cure stage (20 min to 180 min). In the first 20 min to 58 min, the viscosity of the epoxy resin decreased and was prone to flow, resulting in an increase in strain. As the curing process continued, the curing shrinkage started and transferred to the FBG sensor. From this time, the strains measured by the FBG sensor began to decrease and the moment was determined as the gel point. It can be seen from Figure 5a that the gelation moments measured by the FBG sensors were consistent, indicating the uniformity of the curing degree and the correctness of the measurement results.

**Figure 6:**
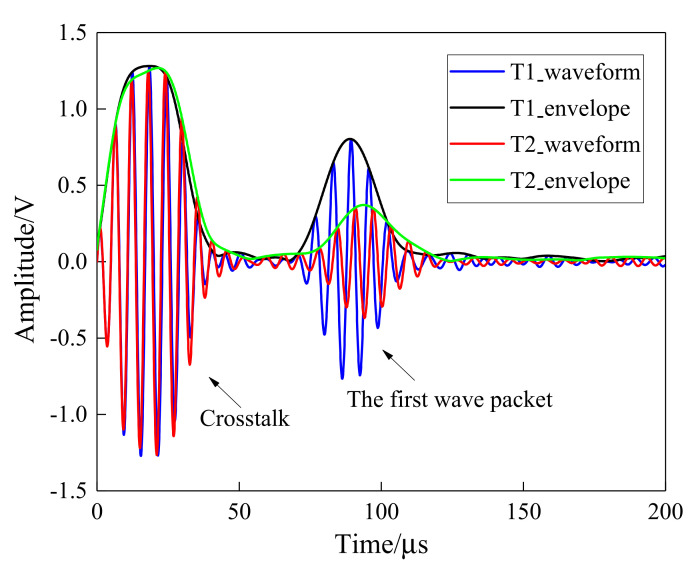
The Hilbert transform results on the received waveforms for one sensing path (T1 = initial moment, T2 = 20 min).

**Figure 7:**
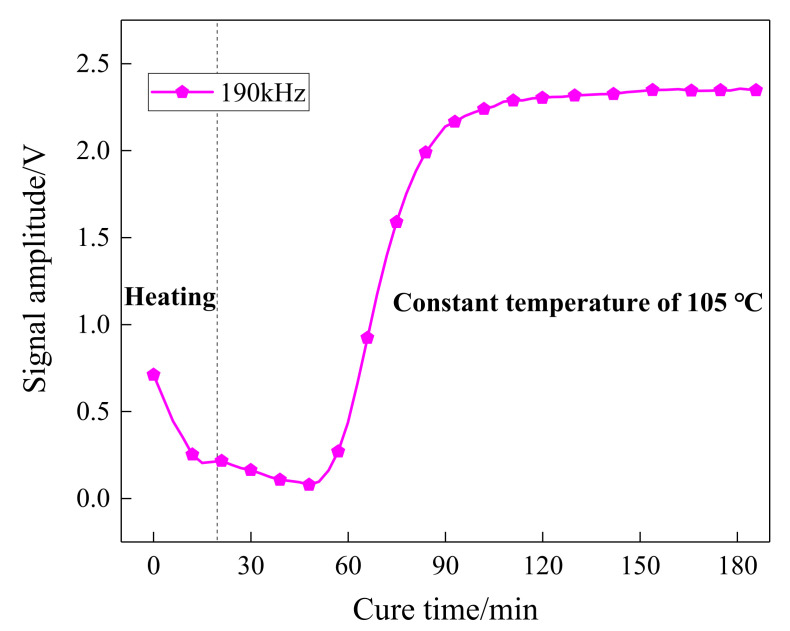
The variation amplitude of the P7_4 sensing path over time.

The amplitude and attenuation of guided waves were obtained by signal processing on the received waveforms. The Hilbert transform, as a reliable signal processing method, is easy to perform and can compensate for the effect of phase difference. Figure 6 shows the Hilbert transform results on the received waveforms at different curing moments of one sensing path. As can be seen, there was a distinct wave packet in the collected signal, which varies with curing time. The amplitude signals of the sensing path P7_4 (PZT 7 serves as an actuator and PZT 4 serves as a sensor) obtained in the experiment was processed and is further shown in Figure 7. The first 20 min was the heating-up stage, and the signal amplitude reduced with the increase in temperature and reached the minimum values. After that, the amplitude started to increase followed by a stable tendency.

**Figure 8:**
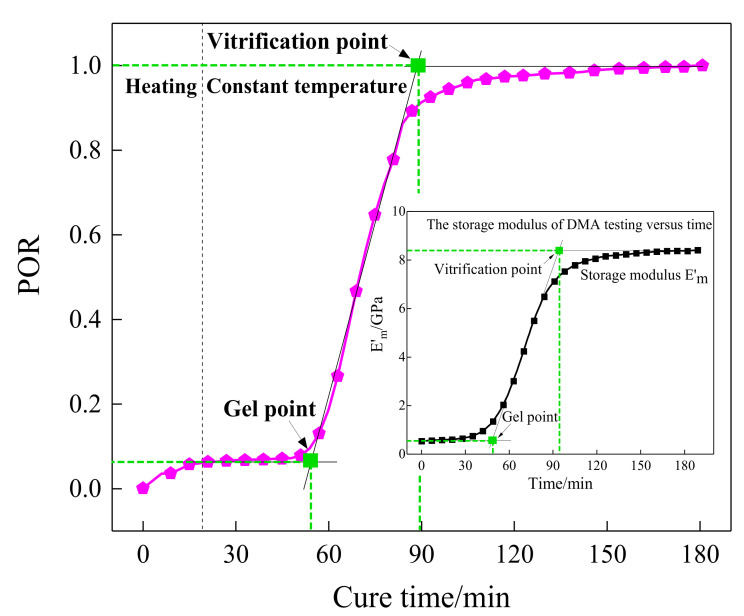
The POR curve of path P7_4 versus curing time.

Considering that the curing of resin is an irreversible and gradual process, the amplitude of path P7_4 was integrated to estimate the progress of the reaction (POR) by the following equation [37]:

(1) $$\int_{t_{1}}^{t_{n}}Xdt\approx \sum_{i=1}^{n-2}\frac{1}{2}(t_{i+1}-t_{i})(\left|x_{i+2}-x_{i+1}\right|+\left|x_{i+1}-x_{i}\right|)$$

where *X* is the integral variable, that is the signal amplitude. $x_{i}$ denotes the amplitude of guided waves at time $t_{i}$. The POR development of the sensing path P7_4 was calculated and is plotted in Figure 8, which demonstrated the changing regularity of the whole curing process. According to Figure 8, the curing process was divided into three sections for analysis. In the initial stage (0–50 min), the temperature rose and the curing reaction had just begun with a slow reaction rate, which is why the POR value increased subtly. As the curing reaction proceeded, the viscosity of the resin increased sharply from 51 min to 90 min, and the POR value increased with a rapid speed accordingly because the cross-linking reaction inside the resin formed a netlike structure. Finally, the POR value continued to increase at a slow rate until it reached the maximum value.

By making a comparison between Figure 8 and the storage modulus curve of the DMA test in Figure 3, it can be observed that the tendency of the POR curve was consistent with the storage modulus variation of the resin, and the first and the second characterization points that occurred in the curing progress were approximately equal to those of DMA results. Consequently, the presented POR curve can approximately characterize the change in storage modulus of the resin under the actual curing environment. To represent the degree of cure, the first and the second characterization points were approximated as the gel point and vitrification point. It can also be seen from the comparison between Figure 5 and Figure 8 that they almost had the same gel point, demonstrating the correctness of the determination of the crucial steps.

**Figure 9:**
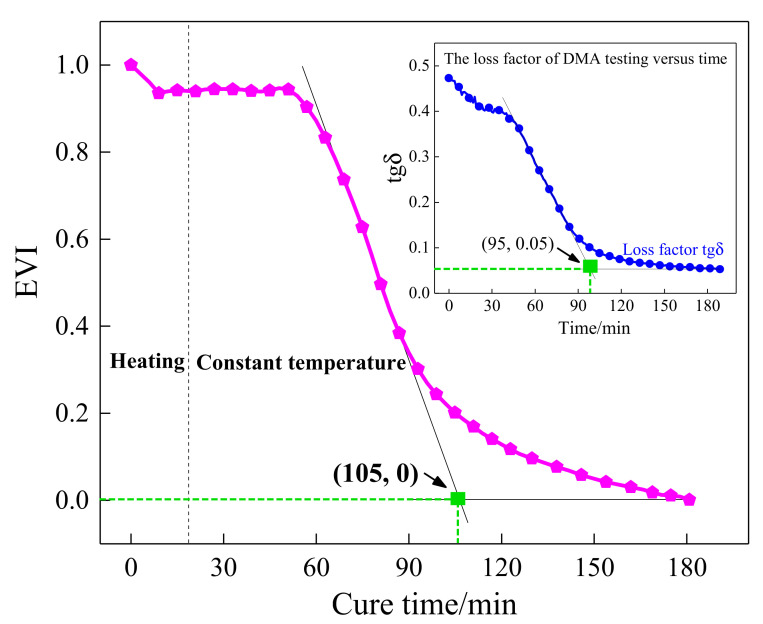
The schematic diagram of the calculated EVI of the sensing path P7_4.

As described in Figure 3, the storage modulus continued to increase versus curing time, but the variation tendency of the loss factor was inverse to that of the storage modulus. As the resin cured, the loss factor continued to decrease until it reached a minimum, which means the continuous reduction in the system energy loss. Therefore, the energy variation index (EVI) can be used to characterize the changes in the system energy during the curing process, which is determined below:

(2) $$\mathrm{EVI}=1-\sqrt{\frac{\int_{t_{b}}^{t_{c}}\left(s_{i}{}^{2}(t)+e_{i}{}^{2}(t)\right)}{\int_{t_{b}}^{t_{c}}\left(b_{i}{}^{2}(t)+c_{i}{}^{2}(t)\right)}}$$

where *t_b_* and *t_c_* are the initial and the end moment of integration, respectively. $b_{i}(t)$ is the signature of the *i-th* sensing path in the initial time, and $c_{i}(t)$ is the Hilbert transform of $b_{i}(t)$. $s_{i}(t)=a_{i}(t)-b_{i}(t)$ is the scattering signal obtained by subtracting the baseline signal $b_{i}(t)$ from the current signal $a_{i}(t)$, and $e_{i}(t)$ is the Hilbert transform of $s_{i}(t)$. Based on the EVI equation, the normalized EVI of the sensing path P7_4 with excitation frequencies of 190 kHz was calculated and is plotted in Figure 9. According to Figure 9, the EVI curve expressed a pronounced downtrend. In the initial stage of curing, the resin had the largest loss modulus corresponding to the maximum EVI value. Then, the energy variation continued to decrease, which means that the curing process nearly completed. The estimated EVI was compared with the loss factor in the DMA test to assess the effectiveness of the proposed characteristic parameter. It can be seen that the characteristic point defined based on the EVI was very close to that determined by the loss factor curve in the DMA test. Hence, the EVI variation was able to assess the loss factor of the resin during the curing process.

**Figure 10:**
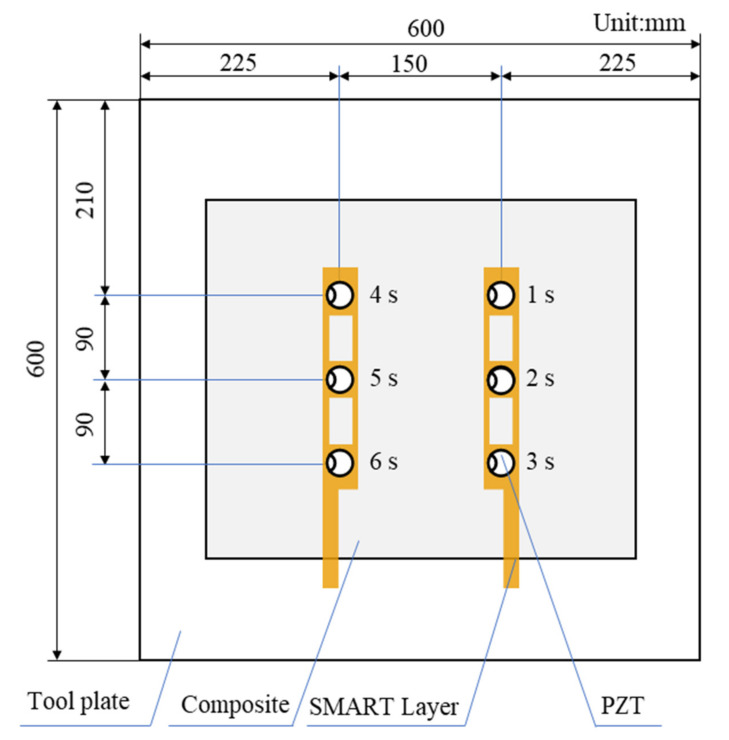
The schematic diagram of the fabricated specimen with layup [0/45/90/−45]_2s_.

**Figure 11:**
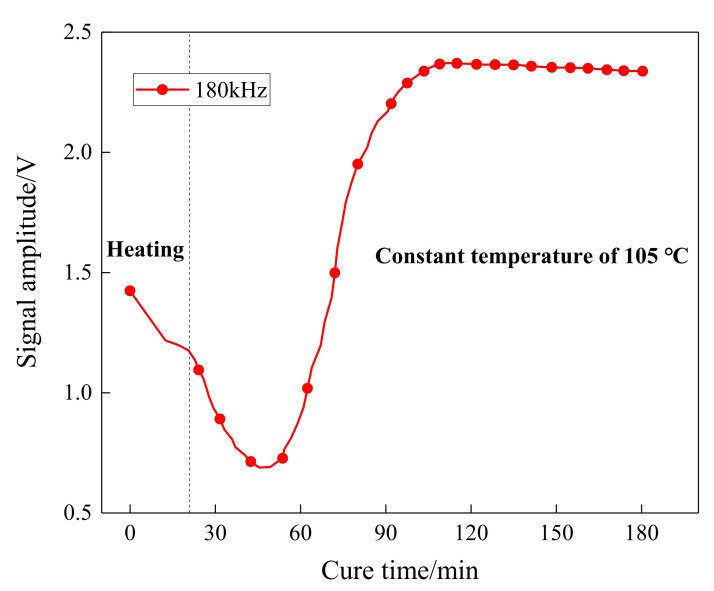
The amplitude variation of the sensing path P4s_6s over time.

**Figure 12:**
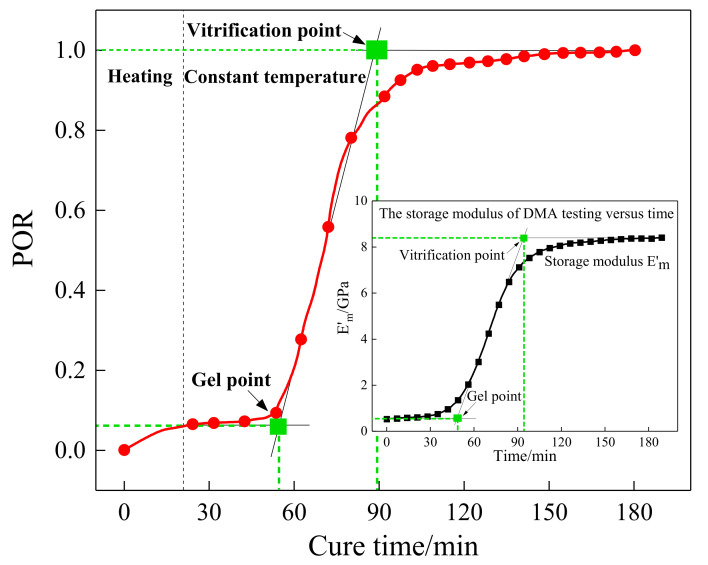
The POR curve of the sensing path P4s_6s for the quasi-isotropic specimen.

**Figure 13:**
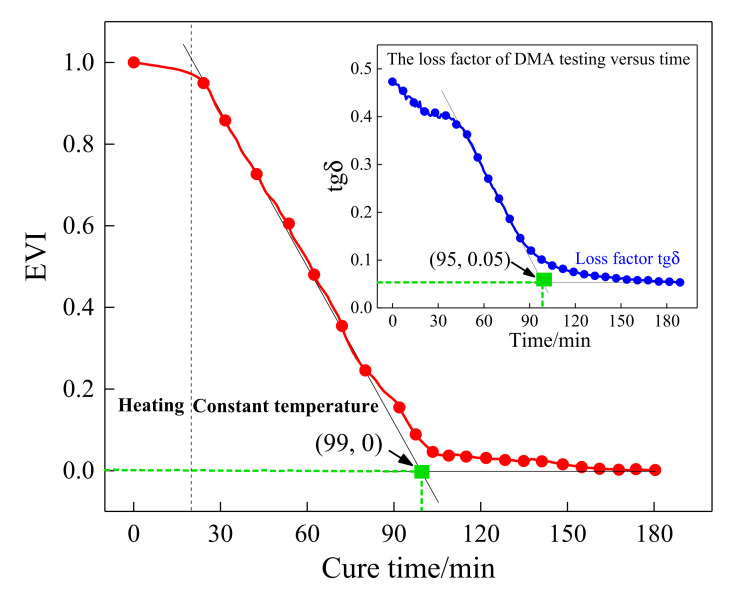
The EVI curve of the sensing path P4s_6s for the quasi-isotropic specimen.

Furthermore, a quasi-isotropic CFRP plate with layup [0/45/90/−45]_2s_ was fabricated and monitored to further validate the effectiveness of the characteristic parameters, including POR and EVI, and the schematic diagram of the specimen is shown in Figure 10. Similarly, the first wave packet of the received waveforms was extracted by the Hilbert transform method, and the processing results are shown in Figure 11. A similar procedure of comparison was made between Figure 7 and Figure 11, as well as Figure 8 and Figure 12. It can be seen that although the guided waves propagated on the CFRP plate with a different lay-up, the amplitude variation had a very similar tendency. With the prolongation of curing time, the amplitude of ultrasonic guided waves decreased first and then increased steadily. For ease of analysis, the POR curve was calculated for the signal amplitude of the sensing path P4 s_6 s using Equation (1), and the variation trend is shown in Figure 12. It can be observed that the approximate gel point and glass transition point that appeared in the POR curve almost coincided with that in the storage modulus curve provided by the DMA test. Similarly, the EVI curve of the sensing path P4 s_6 s for the quasi-isotropic specimen and the loss factor of the DMA test have been merged into Figure 13 for comparison. It can also be easily discovered that the EVI curve expressed a high similarity to the DMA result. The variation tendency indicated that the variation in storage modulus and loss factor in the curing progress can be effectively assessed by the proposed parameters of POR and EVI, respectively. Moreover, several key steps including the gel point and glass transition point can also be correctly identified with the help of the POR curve.

It can be known from the above experimental results that the proposed hybrid piezoelectric-fiber sensor network was integrated with composite structures during the VBM process and can be used to monitor the progress of the reaction, storage modulus, and loss factor of resin, strain, and the crucial steps. It should be noted that both POR and the development of the storage modulus of resin had a great impact on the performance of composite structures and can be used to represent the degree of cure of the resin. The higher the degree of cure, the more the mechanical properties of the fabricated products can be fully exploited. In addition, the development of POR and storage modulus can identify the chemo-physical transformation of the curing matrix for revealing physical phenomena inside materials, such as gelation and vitrification. The determination of each step by the method proposed in this paper can provide a data basis for improving the curing process.

## 3. Service Monitoring

**Figure 14:**
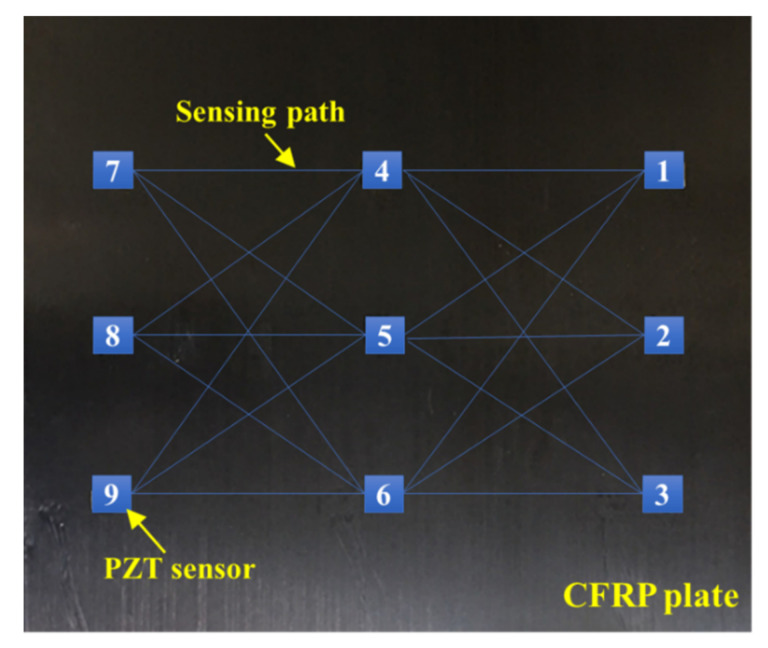
The sensing paths of the fabricated CFRP plate.

After the CFRP is demolded, the embedded piezoelectric-fiber hybrid sensor network can be permanently integrated with the structure to perform in situ and real-time structural health monitoring applications as it makes the composite part “smart”. In this section, damage identification and location were performed to assess the effectiveness of the embedded PZT sensor network, and a modified probability imaging method was proposed to deal with the disadvantage of the conventional PDI method and to improve the accuracy of damage imaging. The damage identification experiments were conducted on the demolded CFRP plate based on the modified PDI method. A simulated damage with a size of ϕ20 mm×2 mm was attached to the CFRP surface at different locations. A diagrammatic sketch of the sensing paths is shown in Figure 14.

### 3.1. Probability Damage Imaging with the Averaged Shape Factor

The PDI method is an imaging method based on the damage index and the weight distribution function to estimate the damage probability of each point in the monitoring area. The damage probability of each pixel can be determined using the equation below [38]:

(3) $$P_{r}(x,y)=\sum_{r=1}^{N}f_{r}\cdot W_{r}[L_{r}(x,y)]$$

where *N* is the number of the transducers for damage identification. $f_{r}$ is the damage-sensitive index of the $r_{th}$ sensing path. The weight distribution function *W_r_* is a function of the relative distance $L_{r}(x,y)$, and can be expressed as:

(4) $$W_{r}[R_{r}(x,y)]=\left\{\begin{array}{ll} 1-\frac{L_{r}(x,y)}{\beta },\; & L_{r}(x,y)<\beta \\ 0, & L_{r}(x,y)\geq \beta \end{array}\right.$$

where β is a shape factor that controls the affected area. $L_{r}(x,y)$ is the relative distance between the pixel (*x*, *y*) and the *r-th* sensing path, which is defined by the following equation:

(5) $$L_{r}(x,y)=\frac{D_{a,r}(x,y)+D_{s,r}(x,y)}{D_{r}}-1$$

in which $D_{r}$ is the distance from the actuator and the transmitter of the *r-th* sensing path. $D_{a,r}(x,y)$ is the distance between the estimated point (*x*, *y*) and the actuator. $D_{s,r}(x,y)$ is the distance between the estimated point (*x*, *y*) and the sensor.

The damage index used in this paper can be written as:

(6) $$DI=\frac{\int_{0}^{T}{\left(C_{r}(t)-B_{r}(t)\right)}^{2}dt}{\int_{0}^{T}B_{r}{}^{2}(t)dt}$$

where $C_{r}(t)$ and $B_{r}(t)$ denote the current signal and baseline signal of the *r-th* sensing path, respectively. *T* is the length of the intercepted signal. From the derivation of Equation (4), it can be found that regardless of whether the damage is located on the direct sensing path, the estimated probability is largest on the sensing path, and it decreases with the distance. Furthermore, the parameter β is empirically chosen and remains constant, which means the same elliptic affected region for all sensing paths. Considering that the sensor configuration and the distance of damage away from each sensing path are different, it is not reasonable for the parameter β to remain unchanged, which would make the imaging results inaccurate or even completely wrong. In order to improve the accuracy of damage location, the range of β was set from 0.05 to 0.3 with a step size of 0.05 in this paper. The imaging associated with multiple values of shape factor β were respectively calculated, and then the results were averaged to obtain the averaged fusion image reconstructed results. It should be noted that in the traditional PDI method, each sensing path only has a single excitation frequency. However, not every excitation frequency is sensitive to the damage. Therefore, it is necessary to select frequencies that are sensitive to damage for service monitoring, which are 50 kHz, 60 kHz, and 70 kHz. Based on the above analysis, a fused PDI method with averaged β and three fusion frequencies was proposed to improve the consequence of one simulated damage imaging.

**Figure 15:**
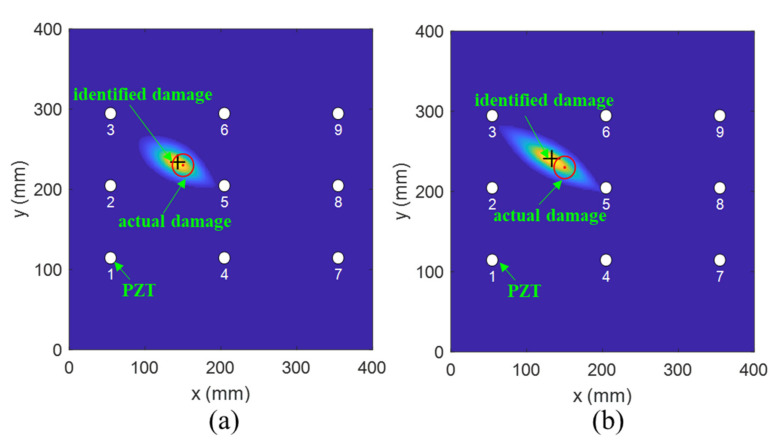
The imaging results of one virtual damage at location (150 mm, 230 mm) obtained by (**a**) the fused PDI method and (**b**) the conventional PDI method.

**Figure 16:**
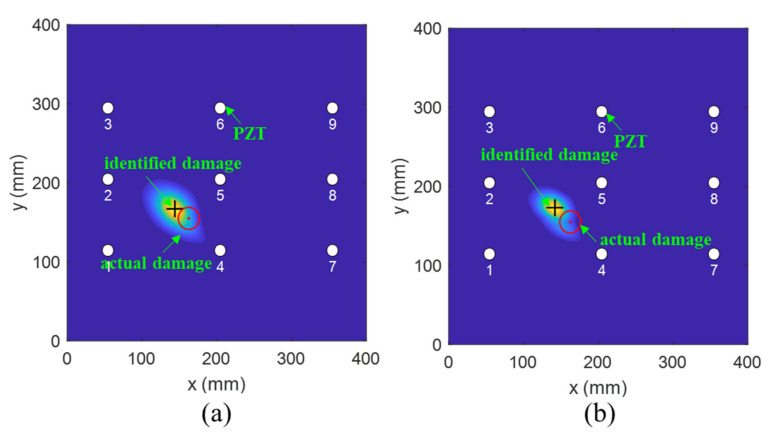
The imaging results of one virtual damage at location (162 mm, 155 mm) obtained by (**a**) the fused PDI method and (**b**) the conventional PDI method.

To investigate the effectiveness of the fused PDI method, a detailed analysis was conducted. Figure 15 and Figure 16 show the comparison results achieved by the fused and the conventional PDI method in the presence of one simulated damage at (150 mm, 230 mm) and (162 mm, 155 mm), respectively. The red circle and the black plus represent the actual damage and the identified damage, respectively. The white circle denotes the PZT transducer. Through observing Figure 15 and Figure 16, it can be easily found that the accuracy of damage detection and identification of the fused PDI method significantly improved compared with the conventional PDI method, indicating the applicability of the proposed method and the embedded PZT sensor network.

### 3.2. Probability Damage Imaging with the Dynamically Adaptive Shape Factor

**Figure 17:**
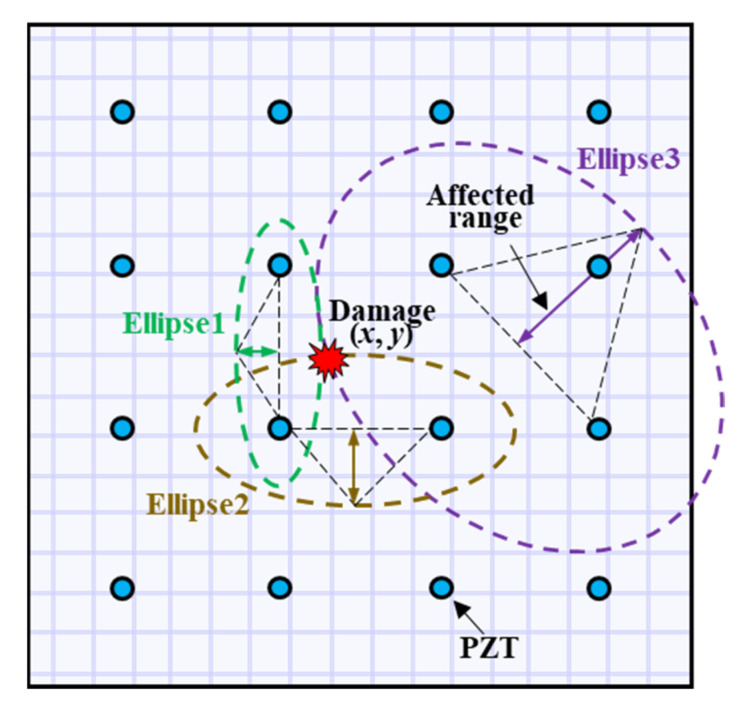
Damage identification based on the PDI method with variable shape factor.

Although the PDI method with the averaged shape factor and multi-frequency fusion can identify the damage location, the image reconstructed results are still unsatisfactory and need to be further improved. As stated above, the traditional way is to select multiple β values, and the imaging results were further fused to improve the imaging accuracy. However, the artificially selected β values may not match each sensing path well. Therefore, it is of great value to dynamically adjust the parameter β of each sensing path to improve the accuracy of damage location. It can be known that the shape factor is used to control the elliptical distribution area affected by the actuator-sensor path. Therefore, it is related to the distance from the damage to the actuator-sensor path, and this distance can be reflected and described by the DI value. Thus, it can be deduced that the shape factor is associated with the DI value and the relationship between the DI and shape factor can be established. It is universally known that when the damage is closer to one sensing path, the damage index corresponding to the sensing path is larger than other sensing paths. Conversely, when the damage index of the sensing path is smaller, it means that the damage is further away from the sensing path. With that in mind, a dynamically adaptive factor $\beta^{adp}$ was proposed to adjust the affected region of each sensing path in real-time based on the damage index. A smaller shape factor was applied to the sensing path with a larger DI because the damage was closer to the current sensing path, while a sensing path with a smaller DI should be imposed with a larger shape factor so that the damage can be included in the affected region, as shown in Figure 17.

**Figure 18:**
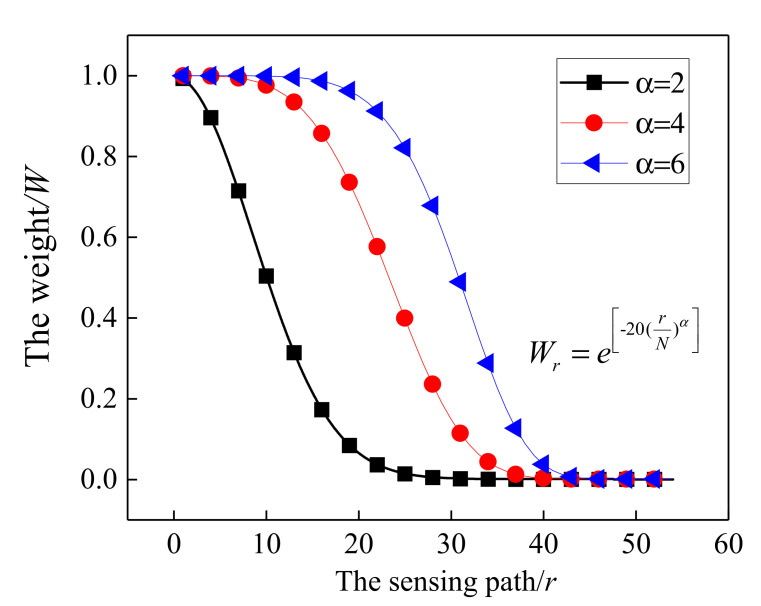
The relationship between the sensing path *r* and the calculated weight *W*.

**Figure 19:**
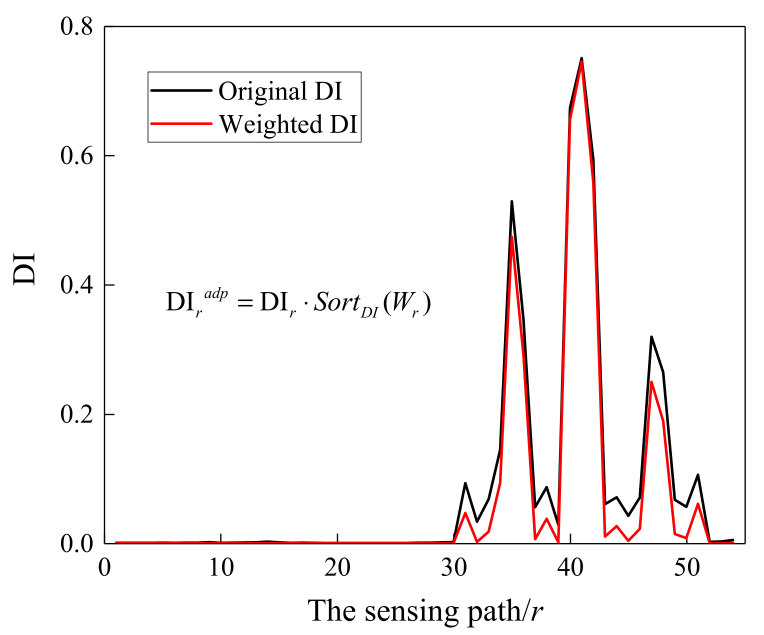
DI variation of all sensing paths before and after applying the weighted factor.

Based on the above analysis, the dynamically adaptive factor $\beta^{adp}$ was negatively correlated with the damage factor of each sensing path. The value of this parameter was smaller than 1.0. However, the minimum value of parameter $\beta^{adp}$ cannot be zero according to Equation (4), and it was set to 0.1 in this paper. Therefore, the dynamically adaptive factor $\beta^{adp}$ can be defined as follows:

(7) $$\beta_{r}{}^{adp}=1-0.9\cdot Norm({\mathrm{DI}}_{r}{}^{adp})$$

in which the symbol of Norm(⋅) means the normalization of DI between 0 and 1. ${\mathrm{DI}}_{r}{}^{adp}$ represents the dynamic damage index of the *r*-*th* sensing path, which is defined as follows:

(8) $$\begin{array}{l} {\mathrm{DI}}_{r}{}^{adp}={\mathrm{DI}}_{r}\cdot Sort_{DI}(W_{r})\\ W_{r}=e^{\left[-20{\left(\frac{r}{N}\right)}^{\alpha }\right]} \end{array}$$

where *N* is the number of the sensing paths. *W_r_* represents the weighting factor that is assigned to the *r-th* path. α is a constant, representing the exponential decay rate of DI. It can be seen from Figure 18 that the relationship between the weighting factor *W_r_* and the sensing path *r* tended to flatten as α increased. Meanwhile, the proportion of larger *W_r_* values increased and the proportion of smaller *W_r_* values decreased, which is not conducive to highlight the actuator-sensor paths associated with the damage. Furthermore, a comparison was conducted on the damage location results when α was set to 2, 4 and 6, and the reconstructed image results showed that it had a higher location accuracy when α was set to 2. $Sort_{DI}(\cdot )$ represents that a specific weight is assigned to the sensing path according to the order of the DI value. By re-sorting, a larger DI corresponds to a larger weight, and a smaller DI has a smaller weight for highlighting the critical path adjacent to damage. The DI variation of all sensing paths before and after applying the weighted factor is shown in Figure 19. It can be seen that the progressive weakening of DI was realized by applying the weighting factor to the corresponding sensing path. Finally, the dynamically adaptive shape factor can be determined by Equation (7).

**Figure 20:**
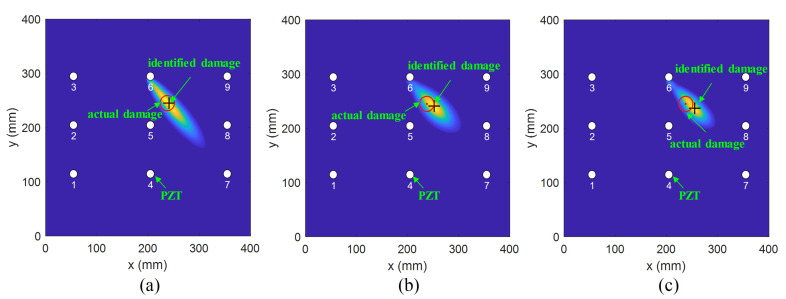
The imaging results of one virtual damage at location (238 mm, 245 mm) obtained by (**a**) the modified PDI method, (**b**) the fused PDI method, and (**c**) the conventional PDI method.

**Figure 21:**
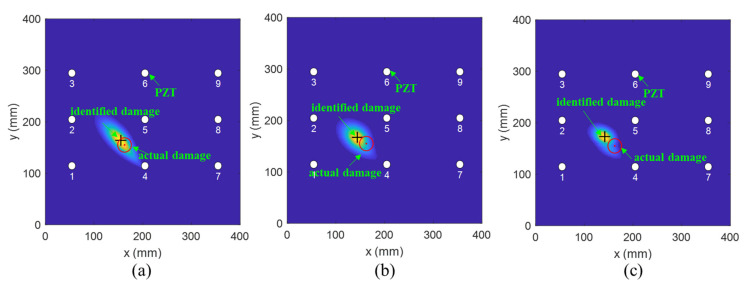
The imaging results of one virtual damage at location (162 mm, 155 mm) obtained by (**a**) the modified PDI method, (**b**) the fused PDI method, and (**c**) the conventional PDI method.

**Figure 22:**
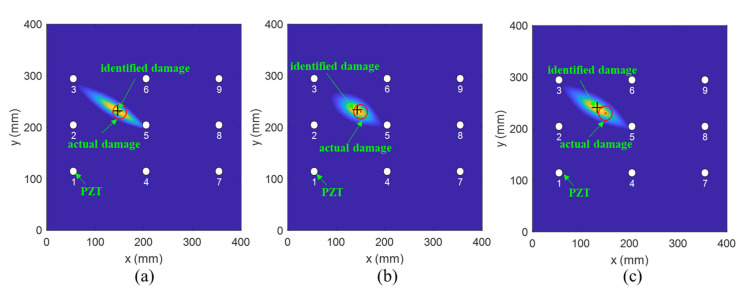
The imaging results of one virtual damage at location (150 mm, 230 mm) obtained by (**a**) the modified PDI method, (**b**) the fused PDI method, and (**c**) the conventional PDI method.

Subsequently, an investigation on damage localization was performed to verify the effectiveness of the modified PDI method. Figure 20, Figure 21 and Figure 22 compare the damage location accuracy of the conventional PDI method, the fused PDI method, and the modified PDI method in the presence of one simulated damage at (238 mm, 245 mm), (162 mm, 155 mm), and (150 mm, 230 mm). It can be seen from the comparison of the three PDI methods that the fused PDI method showed a better prediction performance in damage location than the conventional PDI method. However, the modified PDI results demonstrated in Figure 20a, Figure 21a and Figure 22a expressed a significant improvement in damage location compared with the other two PDI methods. To quantify the damage location accuracy of the three PDI methods, the Euclidean distance and absolute error (AE) between the identified damage and the actual damage were calculated and are enumerated in Table 2. The Euclidean distance was used to calculate the relative distance error (RDE) between the damage center and the center predicted by the imaging method. AE is also a good statistic indicator for measuring the performance of an imaging algorithm, reflecting the deviation in the predicted value from the true value. According to Table 2, it can be deduced that the proposed PDI method had a great improvement in damage location accuracy compared with the conventional PDI method. For virtual damage at location (238 mm, 245 mm), the RDE value of the modified PDI method was 6 mm, which was 50.70% and 63.94% lower than the fused PDI method and the conventional PDI method, respectively. Furthermore, the AE value of the modified PDI method was 50% and 55% lower than those of the other two PDI methods. Similarly, it is easy to observe from Table 2 that the modified PDI method had the minimum AE and RDE, indicating the superior performance and applicability of the modified PDI algorithm in damage identification and the effectiveness of the proposed dynamically adaptive shape factor. Furthermore, the damage imaging results also showed that the embedded PZT sensor network can continuously perform real-time structural health monitoring for the fabricated composite material.

## 4. Conclusions

In this paper, a piezoelectric-fiber hybrid sensor network was developed to perform life-cycle monitoring for composite materials manufactured by the VBM technology. The PZT sensor network and the distributed FBG sensor were inserted inside the composite structures during the manufacturing process to monitor the curing parameters. Furthermore, the PZT sensor network was further used to perform damage identification for composite structures after curing. During the curing process, the signals of guided waves propagating in a plate structure composed of a tool plate, CFRP, release films, and vacuum bag were measured. The amplitude of the first wave packet was obtained by applying the Hilbert transform to the received waveforms. A sharp increase in amplitude could be observed after gelation. In the curing duration, changes in POR and EVI were synchronized with the development of the storage modulus and the loss factor of the resin measured by the DMA test. It first decreased with the temperature and then increased followed by a stable tendency. Furthermore, the distributed FBG sensor was embedded into the unidirectional CFRP to identify the gel point. By observing the strain development, it was found that the gelation time was approximately equal to those of DMA and POR results, indicating the correctness of gelation measurement. The real-time monitoring of the resin curing process and storage modulus could be achieved with the POR curves, and that of the loss factor could be achieved with the EVI curves.

After the curing process was complete, the piezoelectric-fiber hybrid sensor network was integrated permanently with the CFRP plate, and online structural health monitoring was achieved by the PZT-based guided waves monitoring technology. Experiments were conducted on the fabricated CFRP plate to investigate the applicability of the embedded PZT sensors. Furthermore, a modified PDI approach was developed to improve the poor damage identification ability of the conventional PDI method. Two achievements were made in improving the performance of the conventional PDI method. At the first step, the conventional PDI method was modified by applying the fusion frequency and averaged β to assess the health status of composite materials. Some experiments performed on the CFRP plate showed that this modification increased the accuracy of damage localization. In the next step, a dynamically adaptive shape factor and the fusion frequency were applied to the conventional PDI method, and the performance of the proposed method in damage location was evaluated. The images’ reconstructed results from the experimental measurement data showed a significant improvement in damage localization ability of the modified PDI method compared with the conventional PDI and fused PDI methods, indicating the effectiveness of the dynamically adaptive shape factor. The average RDE of the modified PDI method was 68.48% and 46.97% lower than those of the conventional PDI and fusion PDI methods, and the average AE of the modified PDI method was also 64.89% and 43.10% lower than those of the other two PDI methods. Consequently, cure monitoring and in-service inspection are expected to be realized by the embedded piezoelectric-fiber hybrid sensor network.

## Figures and Tables

**Table 1:** The characteristic properties of circular PZT sensors.

Items.	Parameters	Values
s_11_^E^ (×10^−12^ m^2^/N)	Compliance coefficient	10.417
s_12_^E^ (×10^−12^ m^2^/N)	In-plane compliance coefficient	−3.333
*η*	Mechanical loss factor	0.025
$\varepsilon_{33}{}^{T}/\varepsilon_{0}$ (Farad/m)	Relative dielectric constant	1920
*δ*	Dielectric loss factor	0.01
d_31 _(C/N)	Piezoelectric strain constant	−200
*ν*	Poisson’s ratio	0.32
ρ (kg/m^3^)	Density	7750
h (×10^−3^ m)	Thickness	0.33
r (×^10−3^ m)	Radius	4

**Table 2:** Comparisons of localization accuracy between different damage imaging algorithms.

Actual Damage	Modified PDI	AE/mm	RDE/mm	Fused PDI	AE/mm	RDE/mm	Conventional PDI	AE/mm	RDE/mm
(150,200)	(149,201)	1	1.41	(151,201)	1	1.41	(141,202)	9	9.22
(238,245)	(241,245)	6	6.00	(252,241)	12	12.17	(255,237)	14	16.64
(162,155)	(155,164)	9	11.40	(144,167)	18	21.63	(142,173)	20	26.91
(275,290)	(274,291)	1	1.41	(276,287)	3	3.16	(283,287)	8	8.54
(250,170)	(260,165)	10	8.25	(264,163)	14	15.65	(268,161)	18	21.09
(125,200)	(127,201)	2	2.23	(128,203)	3	4.243	(133,204)	8	8.94
(150,230)	(146,232)	4	4.472	(143,234)	7	8.062	(133,241)	17	20.25

## Data Availability

The data presented in this study are available in the article.
